# Taxonomy and evolution of *Aspergillus*, *Penicillium* and *Talaromyces* in the omics era – Past, present and future

**DOI:** 10.1016/j.csbj.2018.05.003

**Published:** 2018-05-31

**Authors:** Chi-Ching Tsang, James Y.M. Tang, Susanna K.P. Lau, Patrick C.Y. Woo

**Affiliations:** a Department of Microbiology, Li Ka Shing Faculty of Medicine, The University of Hong Kong, Hong Kong; b Research Centre of Infection and Immunology, The University of Hong Kong, Hong Kong; c State Key Laboratory of Emerging Infectious Diseases, The University of Hong Kong, Hong Kong; d Carol Yu Centre for Infection, The University of Hong Kong, Hong Kong; e Collaborative Innovation Centre for Diagnosis and Treatment of Infectious Diseases, The University of Hong Kong, Hong Kong

**Keywords:** *Aspergillus*, *Penicillium*, *Talaromyces*, Classification, Evolution, Omics

## Abstract

*Aspergillus*, *Penicillium* and *Talaromyces* are diverse, phenotypically polythetic genera encompassing species important to the environment, economy, biotechnology and medicine, causing significant social impacts. Taxonomic studies on these fungi are essential since they could provide invaluable information on their evolutionary relationships and define criteria for species recognition. With the advancement of various biological, biochemical and computational technologies, different approaches have been adopted for the taxonomy of *Aspergillus*, *Penicillium* and *Talaromyces*; for example, from traditional morphotyping, phenotyping to chemotyping (e.g. lipotyping, proteotypingand metabolotyping) and then mitogenotyping and/or phylotyping. Since different taxonomic approaches focus on different sets of characters of the organisms, various classification and identification schemes would result. In view of this, the consolidated species concept, which takes into account different types of characters, is recently accepted for taxonomic purposes and, together with the lately implemented ‘One Fungus – One Name’ policy, is expected to bring a more stable taxonomy for *Aspergillus*, *Penicillium* and *Talaromyces*, which could facilitate their evolutionary studies. The most significant taxonomic change for the three genera was the transfer of *Penicillium* subgenus *Biverticillium* to *Talaromyces* (e.g. the medically important thermally dimorphic ‘*P. marneffei*’ endemic in Southeast Asia is now named *T. marneffei*), leaving both *Penicillium* and *Talaromyces* as monophyletic genera. Several distantly related *Aspergillus*-like fungi were also segregated from *Aspergillus*, making this genus, containing members of both sexual and asexual morphs, monophyletic as well. In the current omics era, application of various state-of-the-art omics technologies is likely to provide comprehensive information on the evolution of *Aspergillus*, *Penicillium* and *Talaromyces* and a stable taxonomy will hopefully be achieved.

## 1 Introduction

*Aspergillus*, *Penicillium* and *Talaromyces* are diverse genera which belong to the Order *Eurotiales* and contain a large number of species possessing a worldwide distribution and a huge range of ecological habitats. They are ubiquitous and can be found in the air, soil, vegetation and indoor environments [1,2]. Some members are able to grow in extreme environments such as those with high/low temperatures, high salt/sugar concentrations, low acidities or low oxygen levels [3,4]. Species of the three genera are mainly environmental saprobes [3,4] and the primary contribution of these microorganisms to nature is the decomposition of organic materials [1].

Many *Aspergillus*, *Penicillium* and *Talaromyces* species are economically, biotechnologically and medically important with huge social impacts. For example, these species are vital to the food industry and quite a number of them are exploited to produce fermented food such as cheeses (e.g. *P. roqueforti*), sausages (e.g. *P. nalgiovense*) and soy sauce (e.g. *A. oryzae* and *A. sojae*). These fungi are also important biotechnologically for their strong degradative abilities which have been utilised for the production of enzymes [5,6]. In addition, they are robust producers of a diverse spectrum of secondary metabolites (or extrolites) some of which could be used as drugs and antibiotics or as the lead compounds of potential drug candidates with pharmaceutical or biological activities [7]. On the other hand, many of these species, such as *A. chevalieri*, *A. flavipes*, *P. citreonigrum* and *T. macrosporus*, are food spoiling decomposers which cause pre- and post-harvest devastation of food crops; and many of these food-spoiling species are also mycotoxin-producers [8]. Even worse, some of them are infectious agents and cause diseases in humans and animals. The most notorious pathogenic species on a global sense is *A. fumigatus* [9], which is the aetiological agent for the majority of aspergillosis cases [10]. Other commonly encountered pathogenic *Aspergillus* species include *A. flavus*, *A. nidulans*, *A. niger* and *A. terrus*. Although *Penicillium* and *Talaromyces* species are less commonly associated with human or veterinary infections, the thermally dimorphic fungus *T. marneffei*, previously known as *P. marneffei*, is an exception. This notorious fungus is endemic in Southeast Asia and it is able to cause systemic infections particularly in immunocompromised individuals such as HIV-positive patients [11] or patients with impaired cell-mediated immunity [12].

*Aspergillus*, *Penicillium* and *Talaromyces* were traditionally classified according to their morphologies. As technologies capable of characterising biological macromolecules advanced, various approaches focusing on the profiles of different cellular constituents such as lipids, proteins and exometabolites have emerged to supplement the taxonomy of these fungi. The availability of DNA sequencing technology in the past two-to-three decades has generated an enormous amount of DNA sequence data, allowing fungal taxonomy through phylogenetics, including genealogical concordance. The currently accepted consolidated species concept [13], or informally known as the ‘polyphasic taxonomic approach’, has revolutionised fungal taxonomy, and the classification scheme for a vast number of fungi has been revised. In particular, significant changes have been made to reclassify *Aspergillus*, *Penicillium* and *Talaromyces* species in the past seven years. Such revision on the classification of these fungi results in redefined species concepts for *Aspergillus*, *Penicillium* and *Talaromyces*, providing new insights on the evolution of these important filamentous fungi. In this article, the development of various taxonomic approaches as well as species recognition and identification schemes for *Aspergillus*, *Penicillium* and *Talaromyces* is reviewed. These include the traditional morphological/phenotypic approach, the supplementary lipidomic, proteomic and metabolomic approaches, as well as the currently widely used phylogenetic/consolidated approach. The clinical implications of this evolving taxonomy are also discussed.

## 2 Classification and nomenclature: a brief history and recent development

**Fig. 1:**
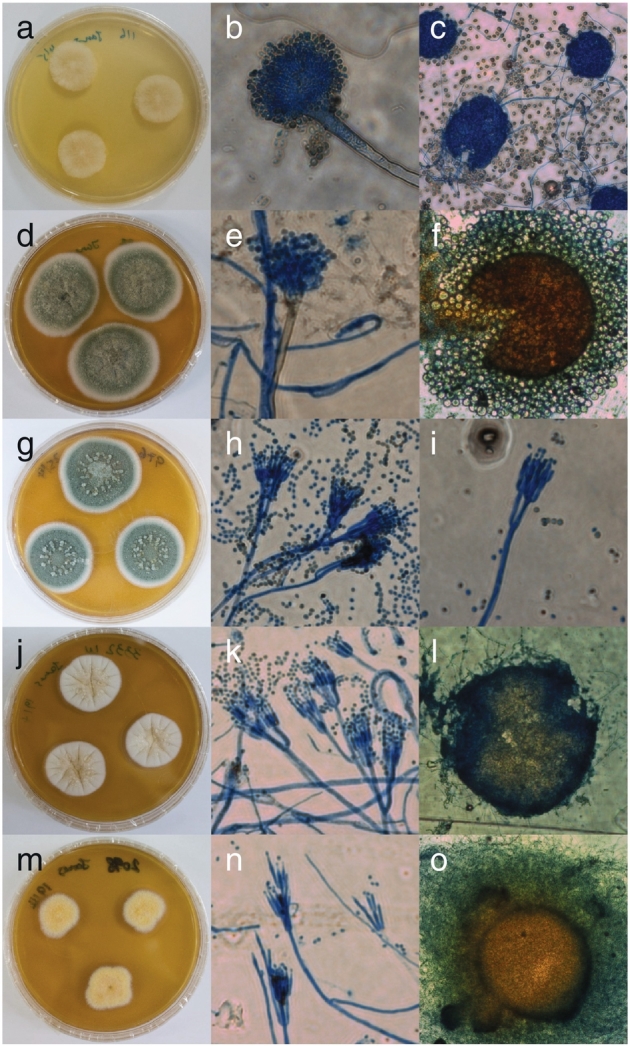
Morphological features of *Aspergillus*, *Penicillium* and *Talaromyces* species. (a) Colony morphology after 7 days of incubation on dichloran 18% glycerol agar, (b) a conidiophore (magnification 400×) and (c) ascomata (*Eurotium*-like sexual stage, magnification 200×) of *A. glaucus* NRRL 116^T^. (d) Colony morphology after 7 days of incubation on malt extract agar (MEA), (e) a conidiophore (magnification 400×) and (f) an ascocarp (*Emericella*-like sexual stage, magnification 100×) of *A. nidulans* NRRL 187^T^. (g) Colony morphology after 7 days of incubation on MEA and (h and i) conidiophores (magnification 400×) of *P. expansum* NRRL 976^T^. (j) Colony morphology after 7 days of incubation on MEA, (k) conidiophores (magnification 400×) and (l) an ascocarp (*Eupenicillium*-like sexual stage, magnification 100×) of *P. kewense* NRRL 3332^T^. (m) Colony morphology after 7 days of incubation on MEA, (n) conidiophores (magnification 400×) and (o) an ascocarp (magnification 100×) of *T. flavus* NRRL 2098^T^.

The name *Aspergillus* was first introduced by Micheli in 1729 to describe asexual fungi whose conidiophores resembled an aspergillum, a device used to sprinkle holy water [14] (Fig. 1a–f). Later in 1768 von Haller validated the genus [15] and in 1832 Fries sanctioned the generic name [16]. Similarly, the genus *Penicillium* was erected by Link in 1809 [17] to accommodate asexual fungi which bore penicillum (painter’s brush)-like fruiting bodies (Fig. 1g–l).

Although both *Aspergillus* and *Penicillium* were originally described as anamorphic (asexual), some species of the two genera were subsequently found to be ascocarp-forming (Fig. 1c, f and l). For example, the sexual genus *Eurotium* was first firmly connected to *Aspergillus* by de Bary in 1854 [18] whereas the ascomycetous genus *Eupenicillium* has been used to describe *Penicillium* species capable of producing sclerotoid cleistothecia from as early as 1892 [19]. Since the discovery of the various sexual states of *Aspergillus* and *Penicillium* species, it has been controversial as to whether separate sexual generic names should be used to describe species able to produce ascospores. In spite of the fact that several sexual genera had already been established to accommodate the sexual morphs of some *Aspergillus* and *Penicillium* species, Thom, Church, Raper and Fennell, in their monographic masterpieces on the taxonomy of these two genera, neglected the use of sexual names. This was because, in their opinions, this would cause unnecessary nomenclatural confusion, especially for strains which were in sexual stages at first and then lost their ascospore-forming ability under laboratory maintenance. In addition, this would also lead to the fragmentation of the large and obviously cohesive *Aspergillus*/*Penicillium* groups [[20], [21], [22], [23], [24], [25]]. Nevertheless, in order to abide by the then *International Code of Botanical Nomenclature* (*Stockholm Code*), where the first valid names of the ‘perfect states’ (sexual morphs) of fungi took precedence [26], Benjamin assigned *Aspergillus* species which possess sexual life cycles into the sexual genera *Eurotium*, *Emericella* and *Sartorya* [27]. In addition, he transferred *Penicillium* species with sexual life cycles to the ascomycetous genus *Carpenteles* (later synonym of *Eupenicillium*) [27,28]. During his assignment, Benjamin also established the novel genus *Talaromyces* to describe *Penicillium* species which, in their sexual life cycles, possessed soft ascocarps exhibiting indeterminate growth and whose walls were composed of interwoven hyphae [27] (Fig. 1m–o).

As the number of species of the genera *Aspergillus*, *Penicillium* and *Talaromyces* increased, closely related species were grouped into subgroups [[29], [30], [31], [32]]. Such infrageneric classification system underwent vigorous changes since different authors focused on different morphological features when establishing their subgrouping schemes (Table 1). For example, Blochwitz as well as Thom and his co-workers were the first to divide *Aspergillus* species into seven and 18 subgeneric ‘groups’, respectively, based on their phenotypes [21,24,30,31]. The subgrouping by Thom and associates formed the foundation of *Aspergillus* subgeneric classification which had been largely followed by other mycologists working on this genus in the last century. However, since these subgeneric ‘groups’ did not possess any nomenclatural status, Gams et al., in 1986, established six subgenera and 18 sections to accommodate these ‘groups’, formalising the subgeneric classification of *Aspergillus* species [33] (Table 1a). As for *Penicillium*, Dierckx and Biourge firstly subdivided the genus into the subgenera *Aspergilloides* (synonym: *Monoverticillium*) as well as *Eupenicillium*, which was further separated into sections *Biverticillium* and *Bulliardium* (synonym: *Asymmetrica*) [29,34]. Subsequently, Thom and his co-workers did not follow Dierckx’s and Biourge’s grouping and proposed a new subgeneric classification scheme for *Penicillium* composed of four main divisions/sections, where species were grouped according to features of their colonies and branching patterns of their conidiophores [20,22]. The system established by Thom and associates for *Penicillium* was adopted by other mycologists for the next 30 years until Pitt as well as Stolk and Samson in the 1980s proposed two other subgeneric classification schemes based on features of conidiophores, morphology of phialides and growth characteristics, as well as branching patterns of conidiophores and phialide morphology, respectively [35,36] (Table 1b). Similarly, *Talaromyces* species were also split into four sections based on the structures of their conidial states [32] (Table 1c).

**Table 1a:** Overview of major subgeneric classifications of *Aspergillus* species.

Blochwitz [31]	Thom & Church [30], Thom & Raper [21], Raper & Fennell [24]	Gams et al. [33]	Peterson [168]	Peterson et al. [169]	Houbraken & Samson [3]	Houbraken et al. [4]	Jurjević et al. [170], Kocsubé et al. [60], Sklenář et al. [171]
*Euglobosi*	Group *A. candidus*	Subgenus *Aspergillus*	Subgenus *Aspergillus*	Subgenus *Aspergillus*	Subgenus *Aspergillus*	Subgenus *Aspergillus*	fSubgenus *Aspergillus*
*Flavi*	Group *A. cervinus*	Section *Aspergillus*	Section *Aspergillus*	Section *Aspergillus*	Section *Aspergillus*	Section *Aspergillus*	Section *Aspergillus*
*Fulvi*	Group *A. clavatus*	Section *Restricti*	Section *Candidi*	Section *Restricti*	Section *Restricti*	Section *Restricti*	Section *Restricti*
*Glauci*	Group *A. cremeus*	Subgenus *Circumdati*	Section *Cervini*	Subgenus *Candidi*	Subgenus *Circumdati*	Subgenus *Circumdati*	Subgenus *Circumdati*
*Nidulantes*	Group *A. flavipes*	Section *Candidi*	Section *Circumdati*	Section *Candidi*	Section *Candidi*	Section *Candidi*	Section *Candidi*
*Nigroides*	Group *A. flavus*	Section *Circumdati*	Section *Cremei*	Subgenus *Circumdati*	Section *Circumdati*	Section *Circumdati*	gSection *Circumdati*
*Phaei*	Group *A. fumigatus*	Section *Cremei*	Section *Flavi*	Section *Circumdati*	Section *Flavi*	Section *Flavi*	hSection *Flavi*
	Group *A. glaucus*	Section *Flavi*	Section *Flavipedes*	Section *Cremei*	Section *Flavipedes*	Section *Flavipedes*	iSection *Flavipedes*
	Group *A. nidulans*	Section *Nigri*	Section *Nigri*	Section *Flavi*	Section *Nigri*	Section *Nigri*	Section *Jani*
	Group *A. niger*	Section *Sparsi*	Section *Restricti*	Section *Nigri*	Section *Terrei*	Section *Terrei*	Section *Nigri*
	Group *A. ochraceus*	cSection *Wentii*	Section *Terrei*	Subgenus *Fumigati*	Subgenus *Fumigati*	Subgenus *Fumigati*	Section *Petersonii*
	aGroup *A. ornatus*	Subgenus *Clavati*	Subgenus *Fumigati*	Section *Cervini*	Section *Cervini*	Section *Cervini*	Section *Robusti*
	Group *A. restrictus*	Section *Clavati*	Section *Clavati*	Section *Clavati*	Section *Clavati*	Section *Clavati*	Section *Tanneri*
	Group *A. sparsus*	Subgenus *Fumigati*	Section *Fumigati*	Section *Fumigati*	Section *Fumigati*	Section *Fumigati*	Section *Terrei*
	Group *A. terreus*	Section *Cervini*	Subgenus *Nidulantes*	aSubgenus *Ornati*	Subgenus *Nidulantes*	Subgenus *Nidulantes*	jSubgenus *Cremei*
	Group *A. ustus*	Section *Fumigati*	aSection *Ornati*	aSection *Ornati*	Section *Aenei*	Section *Aenei*	Subgenus *Fumigati*
	bGroup *A. versicolor*	aSubgenus *Ornati*	Section *Nidulantes*	Subgenus *Nidulantes*	Section *Ochraceorosei*	Section *Bispori*	Section *Cervini*
	cGroup *A. wentii*	aSection *Ornati*	Section *Sparsi*	Section *Bispori*	Section *Nidulantes*	Section *Cremei*	kSection *Clavati*
		Subgenus *Nidulantes*		Section *Ochraceorosei*	Section *Sparsi*	Section *Ochraceorosei*	lSection *Fumigati*
		Section *Flavipedes*		Section *Nidulantes*	Section *Usti*	Section *Nidulantes*	Subgenus *Nidulantes*
		Section *Nidulantes*		Section *Raperi*	Unassigned section	Section *Silvati*	mSection *Aenei*
		Section *Terrei*		Section *Silvati*	Section *Cremei*	Section *Sparsi*	Section *Bispori*
		Section *Usti*		Section *Sparsi*		Section *Usti*	Section *Cavernicolus*
		bSection *Versicolores*		Section *Usti*			Section *Ochraceorosei*
				Subgenus *Terrei*			mSection *Nidulantes*
				Section *Flavipedes*			Section *Raperi*
				Section *Terrei*			Section *Silvati*
				Subgenus *Warcupi*			Section *Sparsi*
				dSection *Warcupi*			mSection *Usti*
				eSection *Zonati*			Subgenus *Polypaecilum*

a Transferred to genus *Sclerocleista* and excluded from *Aspergillus* [3,53]

b Merged with section *Nidulantes* [168]

c Merged with section *Cremei* [172]

d Transferred to genus *Warcupiella* and excluded from *Aspergillus* [3,53]

e Trasnferred to genus *Penicilliopsis* and excluded from *Aspergillus* [3,60]

f Sexual synonym = *Eurotium* [4]

g Sexual synonym = *Neopetromyces* [4]

h Sexual synonym = *Petromyces* [4]

i Sexual synonym = *Fennellia* [4]

j Sexual synonym = *Chaetosartorya* [4]

k Sexual synonym = *Dichotomomyces* and *Neocarpenteles* [4]

l Sexual synonym = *Neosartorya* [4]

m Sexual synonym = *Emericella* [4]

**Table 1b:** Overview of major subgeneric classifications of *Penicillium* species.

Dierckx [29]	Biourge [34]	Thom [20]	Raper et al. [22]	Pitt [35]	Stolk & Samson [36]	Houbraken & Samson [3], Houbraken et al. [173]
Subgenus *Aspergilloides*	aSubgenus *Eupenicillium*	Division *Asymmetrica*	Section *Asymmetrica*	Subgenus *Aspergilloides*	Section *Aspergilloides*	Subgenus *Aspergilloides*
aSubgenus *Eupenicillium*	bSection *Biverticillium*	Section *Brevi-compacta*	bSection *Biverticillata-symmetrica*	Section *Aspergilloides*	bSection *Biverticillium*	Section *Aspergilloides*
	Section *Bulliardium* (=Section *Asymmetrica*)	Section *Fasciculata*	Section *Monoverticillata*	Section *Exilicaulis*	Section *Coremigenum*	Section *Charlesii*
	Subgenus *Monoverticillium*	Section *Funiculosa*	Section *Polyverticillata-symmetrica*	bSubgenus *Biverticillium*	Section *Divaricatum*	Section *Cinnamopurpurea*
		Section *Lanata-divaricata*		Section *Coremigenum*	Section *Eladia*	Section *Citrina*
		Section *Lanata-typica*		Section *Simplicium*	Section *Geosmithia*	Section *Exilicaulis*
		Section *Velutina*		Subgenus *Furcatum*	Section *Inordinate*	Section *Fracta*
		bDivision *Biverticillata-symmetrica*		Section *Divaricatum*	Section *Ramosum*	Section *Gracilenta*
		Section *Ascogena*		Section *Furcatum*	Section *Penicillium*	Section *Lanata-divaricata*
		Section *Coremigena*		Subgenus *Penicillium*	Section *Torulomyces*	Section *Ochrosalmonea*
		Section *Luteo-virida*		Section *Coronatum*		Section *Ramigena*
		Section *Miscellanea*		Section *Cylindrosporum*		Section *Sclerotiora*
		Division *Monoverticillata*		Section *Inordinate*		Section *Stolkia*
		Section *Monoverticillata-stricta*		Section *Penicillium*		Section *Torulomyces*
		Section *Monoverticillata-Ramigena*				Section *Thysanophora*
		Division *Polyverticillata-symmetrica*				Subgenus *Penicillium*
						Section *Brevicompacta*
						Section *Canescentia*
						Section *Chrysogena*
						Section *Digitata*
						Section *Eladia*
						Section *Fasiculata*
						Section *Osmophila*
						Section *Paradoxa*
						Section *Penicillium*
						Section *Ramosa*
						Section *Robsamsonia*
						Section *Roquefortorum*
						Section *Turbata*

a Not referring to the sexual genus *Eupenicillium* Ludwig

b Transferred to genus *Talaromyces* and excluded from *Penicillium*

**Table 1c:** Overview of major subgeneric classifications of *Talaromyces* species

Stolk & Samson [32]	Yaguchi et al. [174]	Yilmaz et al. [63]
aSection *Emersonii*	aSection *Emersonii*	Section *Bacillispori*
Section *Purpurea*	Section *Purpurea*	Section *Helici*
Section *Talaromyces*	Section *Talaromyces*	Section *Islandici*
bSection *Thermophila*	bSection *Thermophila*	Section *Purpurei*
	Section *Trachyspermus*	Section *Talaromyces*
		Section *Subinflati*
		Section *Trachyspermi*

a Transferred to genus *Rasamsonia* and excluded from *Talaromyces* [175]

b Transferred to genus *Thermomyces* and excluded from *Talaromyces* [4]

**Fig. 2:**
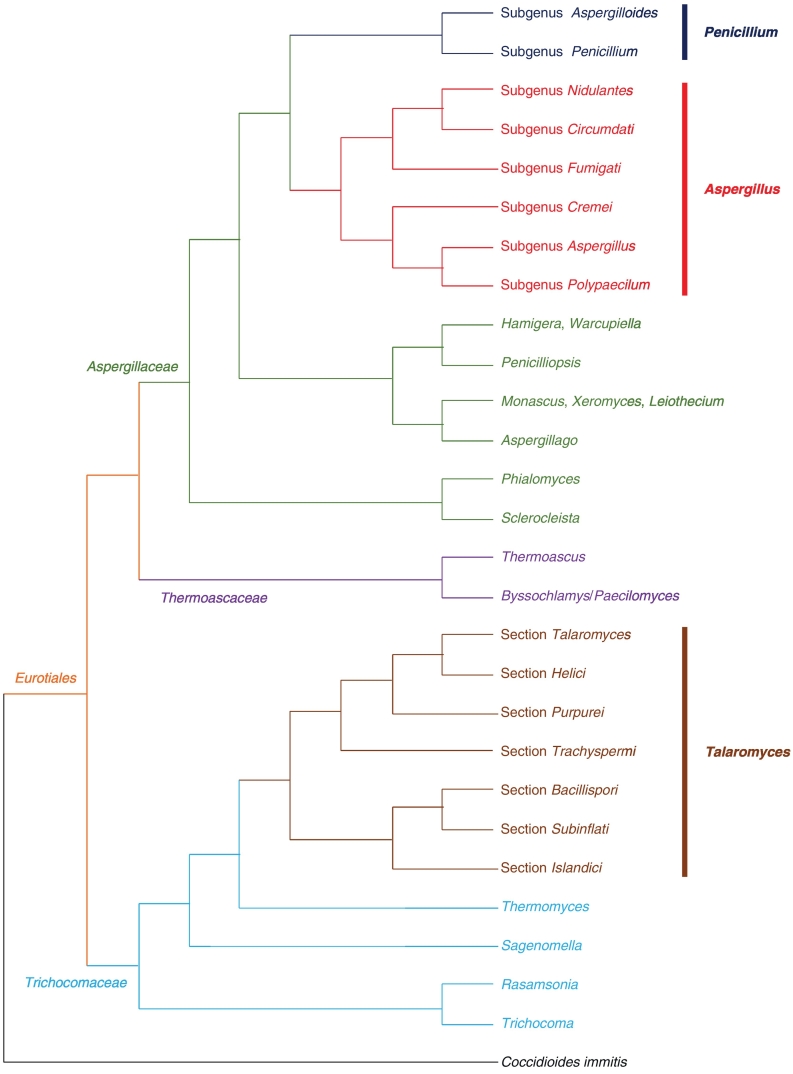
Schematic representation of the phylogenetic relationship, as inferred by Houbraken & Samson [3], Yilmaz et al. [63] and Kocsubé et al*.* [60], amongst members of the order *Eurotiales*. *Aspergillus* and *Penicillium* are sister genera of the family *Aspergillaceae* whereas *Talaromyces* is more distantly related to those two genera and belongs to a separate family, *Trichocomaceae*.

As the species concept for fungi migrates from morphological, physiological, or phenotypic to genetic, phylogenetic (including genealogical concordance) and even consolidated, further changes have been made to the infrageneric classification of *Aspergillus*, *Penicillium* and *Talaromyces* (Table 1). The adoption of the consolidated species concept, with reduced emphasis on morphological properties, in classifying species of these genera resulted in the fact that fungi with aspergillum-shaped conidiophores no longer necessarily are *Aspergillus* species, while fungi with penicillum-shaped conidiophores no longer necessarily are *Penicillium* species [37]. One notable change in relation to these genera, also as a result of the recent implementation of the single-naming system(‘One Fungus – One Name’ [1F1N] principle) [[38], [39], [40]], was the transfer of fungi belonging to *Penicillium* subgenus *Biverticillium* to the genus *Talaromyces* [41], whose close chemotaxonomic relationship [42] and phylogenetic connection [[43], [44], [45]] have been recognised since the 1990s, leaving both the genera *Penicillium* and *Talaromyces* as monophyletic clades [41] (Fig. 2). Interestingly, during this transfer the species *P. aureocephalum* (synonym for sexual morph: *Lasioderma flavovirens*) [46] was also accommodated in the *Talaromyces* clade. Inclusion of this species, which is also the type and only species of the genus *Lasioderma* [47], necessitated the renaming of the *Talaromyces* clade as *Lasioderma*, since this is an older sexual name with nomenclatural priority [48]. However, such renaming would require many name changes (from *Talaromyces* species to *Lasioderma* species) and several species are better scientifically and economically well-known with their *Talaromyces* names. Also, even though using identical names for botanical/mycological and zoological genera is not forbidden by the *Melbourne Code*, the name *Lasioderma* [*Ascomycota*] is a later homonym to *Lasioderma* [Arthropoda] currently in use for one of the beetle genera and this might cause confusion to non-taxonomists. Hence, it was proposed to conserve the generic name *Talaromyces* over *Lasioderma* (*Ascomycota*) [49]. Recently, this proposal was approved by both the Nomenclature Committee for Fungi (NCF) [50] and General Committee for Nomenclature [51] of the International Association for Plant Taxonomy, retaining the generic name *Talaromyces*.

Despite the fact that the taxonomy of *Penicillium* and *Talaromyces* seems straight-forward now since both of them clearly represent two separate monophyletic groups [41], the scenario for *Aspergillus* is much more complicated, involving two opposing generic concepts, namely the wide and narrow *Aspergillus* concepts. Early work by Benjamin summarised the links between *Aspergillus* and the sexual genera *Emericella*, *Eurotium* and *Neosartorya* (erroneously as *Sartorya* by Benjamin which was later found that the original description of *Sartorya* was based on a contaminant in an *A. fumigatus* culture receiving radium radiation) [24,27,52]. Following other subsequent changes in *Aspergillus* classification, seven additional sexual genera, including *Chaetosartorya* [53], *Cristaspora* [2], *Dichotomomyces* [2,54], *Fennellia* [55], *Neocarpenteles* [56], *Neopetromyces* [57] and *Petromyces* [52], are further connected to *Aspergillus*. Remarkably, each of these sexual genera only associates with a particular *Aspergillus* subgenus or section (Table 1a). Subsequent to the adoption of 1F1N, there have been disputes as to whether the generic name *Aspergillus* should be retained for the large monophyletic clade, although weakly supported (~50–70% bootstrap only) by maximum likelihood analyses [3,4], of classical *Aspergillus* species (broad/wide *Aspergillus* concept) [2]; or to adopt sexual names for those well-supported clades containing both pleomorphic species and asexual species with *Aspergillus* morphologies (narrow *Aspergillus* concept; i.e. subgenus *Aspergillus* = *Eurotium*, subgenus *Cremei* = *Chaetosartorya*, subgenus *Fumigati* = *Neosartorya* and subgenus *Nidulantes* = *Emericella*), leaving the weakly supported (<50% bootstrap) [3] subgenus *Circumdati* as *Aspergillus sensu stricto*, even though this group does include several less well-known sexual genera (*Fennellia*, *Neopetromyces* and *Petromyces*) [58]. The latter proposal was advocated based on the fact that the sexual genera *Chaetosartorya*, *Emericella*, *Eurotium* and *Neosartorya* differ significantly in their morphologies, physiologies, enzymologies, as well as toxicologies [59]. Also, Pitt, Taylor and Göker, proposers of the narrow *Aspergillus* concept, found in their phylogenetic analyses that classical *Aspergillus* was paraphyletic, encompassing the monophyletic *Penicillium* clade. As a result, according to Pitt et al. if the wide *Aspergillus* concept is to be adopted then *Pencillium* would also need to be synonymised under *Aspergillus* to make the whole clade monophyletic [58,59]. On the other hand, the main problem for the narrow *Aspergillus* concept rests in the retypification by conservation of the genus. This is because under the narrow *Aspergillus* concept, the type of the genus *Aspergillus*, *A. glaucus* of subgenus *Aspergillus*, would fall in the genus *Eurotium* instead. Since taxonomic properties of the type and related species determine the circumscription of the genus, if the name *Aspergillus* is to be applied to subgenus *Circumdati*, the type of the genus has to be changed to one of the species within this subgenus, for example, *A. niger* as suggested by Pitt and Taylor because of its more frequent use in literatures and databases [58]. However, in the eyes of the wide *Aspergillus* concept advocates, such generic retypification is debatable since the new type of choice would depend on the interest of different fields. For instance, *A. flavus* would be the type of choice for food mycology and mycotoxicology, *A. fumigatus* for medical mycology, whereas *A. nidulans* for fungal molecular genetics [2]. Recently, regarding the narrow *Aspergillus* proposal which considers *Aspergillus* to be non-monophyletic and recommends to apply the name *Aspergillus* only to members of the subgenus *Circumdati* through retypification by conservation while maintaining the sexual names for other supported clades [58,59], Kocsubé et al., supporters of the wide *Aspergillus* concept, demonstrated in their phylogenetic analyses, based on six and nine genetic markers using both maximum likelihood and Bayesian approaches as well as extrolite profiling, that *Aspergillus* represents a well-supported monophyletic clade sister to the monophyletic *Penicillium* clade (Fig. 2) [60], rejecting Pitt et al.’s hypotheses and proposal. They also established the subgenus *Polypaecilum* to encompass species previously assigned to the genera *Phialosimplex* and *Polypaecilum* (Fig. 2), whereas the species *A. clavatoflavus* and *A. zonatus*, which are actually phylogenetically distantly related to *Aspergillus*, were transferred to the novel genera *Aspergillago* as *Aspergillago clavatoflava* and *Penicilliopsis* as *Penicilliopsis zonata*, respectively [60]. Nevertheless, Pitt and Taylor have submitted a formal proposal to the NCF to retypify *Aspergillus* with *A. niger* to redefine the genus to members of subgenus *Circumdati* only, with sexual names taken up to replace other subgeneric names of *Aspergillus* [61]. In response to Pitt and Taylor, Samson et al. urged the NCF to reject the conservation proposal based on their arguments that *Aspergillu*s is monophyletic as well as clearly-defined by phenotypic synapomorphies and secondary metabolite chemistry; that the size of the genus *Aspergillus* is irrelevant; and that conservation with a different generic type (*A. niger*) would lead to unpredictable name changes and would not result in a more stable nomenclature [62]. Recently, voting was held by the NCF and the proposal by Pitt and Taylor could not obtain a 60% majority for the ‘yes’ vote after two rounds of ballots. Although the ‘no’ vote was also one vote short of reaching 60%, it was in the majority. Since there is no definite recommendation from the NCF, this proposal will be referred to the General Committee on Nomenclature for final decision (Dr Tom May, personal communication).

## 3 Species recognition/identification and current advances

Since the establishment of *Aspergillus*, *Penicillium* and *Talaromyces*, species in these genera had been recognised by their morphological features until the dawn of molecular systematics. In particular, morphology of conidial structures, especially their branching patterns as discussed above, has played an important role in species recognition and identification. Other important morphological properties useful for diagnosing a species include cleistothecium and ascus/ascospore (when present) characters [1,2]. Macroscopically, characteristics of the colony, such as texture, growth rate, degree of sporulation, conidial and mycelial colours, as well as production of diffusing pigments, exudates, acids and other secondary metabolites, are also used for species differentiation [1,2,63]. The need for standardisation of culture media and incubation condition for reproducible species identification was recognised as early as Biourge's and Dierckx's time [64]. This is because variations in the immediate cultural environment, such as nutrient availability, temperature, light intensity (including ultraviolet light), water activity, humidity and/or other environmental factors, regardless how subtle these discrepancies are, could change the appearance of the organism since morphology is one of the way in which an organism adapts to and survives in its environment [1]. The effects of these changes in incubation condition have been exemplified by the work by Okura et al. [65,66]. As such, standardised working techniques for morphological characterisation have been recommended for *Aspergillus* and *Penicillium* species [1,2]. Although no standard is proposed for *Talaromyces*, these methods should also be applicable to this genus since by tradition quite a number of *Talaromyces* species were considered and characterised as *Penicillium* species.

With the availability of newer techniques, such as gas–liquid chromatography and electrophoresis, for the characterisation of biomolecules in the 20th century, chemotaxonomy has gained popularity in *Aspergillus*, *Penicillium* and *Talaromyces* taxonomy, especially since the 1980s. One of the approaches for chemotaxonomy is zymogram profiling, where species are differentiated based on the polyacrylamide gel-electrophoretic patterns of certain isoenzymes [67]. This technique has been demonstrated to be highly successful in differentiating species of *Penicillium* subgenus *Penicillium*, where the isozyme patterns showed a high correlation with morphological species [68,69]. However, when species from other *Penicillium* subgenera were also included in the analysis it was found that correlation between zymogram grouping and morphological species only existed in some cases [70], rendering the utility of this technique for the identification of *Penicillium* species questionable. On the other hand, zymogram profiling has also been applied to *Aspergillus* species and this identification method was found to be practical especially for members of the subgenera *Circumdati*, *Fumigati* and *Nidulantes* [[71], [72], [73]], in spite of the fact that some closely related species, such as the wild type *A. flavus* and the domesticated counterpart *A. oryzae* or the wild type *A. parasiticus* and the domesticated *A. sojae*, produced very similar isoenzyme patterns and could not be well differentiated [71]. Nonetheless, fingerprinting of isozymes has not been widely employed as a practical identification system since the enzyme profiles for the vast majority of *Aspergillus*, *Penicillium* and *Talaromyces* species remained uncharacterised. Also, there is no consensus as to which isoenzymes should be used for comparison.

Another approach for chemotaxonomy is extrolite profiling. The exometabolome reflects the physiology of an organism in response to its biotic and abiotic environment [74] and profiling of the exometabolome is particularly useful for the chemotaxonomy of *Aspergillus*, *Penicillium* and *Talaromyces* species since these genera are the best known exometabolite producers, having the most diverse spectra of exometabolites amongst 26 different groups of ascomycetes analysed, which represented four different Classes (*Dothideomycetes*, *Eurotiomycetes*, *Leotiomycetes* and *Sordariomycetes*) [37]. Amongst the various kinds of exometabolites, such as excessive organic acids, extracellular enzymes and accumulated carbohydrates, the one that generally displays more pronounced chemoconsistency and higher species specificity is secondary metabolites [74]. The first insight of the taxonomic value of secondary metabolite profiling was gained when Ciegler et al. attempted to divide *P. viridicatum* into three subgroups, in which the production of the mycotoxins citrinin, ochratoxin, viomellein and xanthomegnin was characterised as one of the classification criteria [75,76]. However, Ciegler et al.’s method required complicated and tedious pre-treatment of the samples. As a result, their approach was only popularised after the development of simpler techniques which only involve direct spotting of small agar plugs from fungal cultures on thin-layer chromatography plates without the need of any preceding extraction or purification procedures [77,78]. Since then, extrolite data have contributed much to species recognition of *Aspergillus*, *Penicillium* and *Talaromyces* species. For example, using secondary metabolite profiling Frisvad and Filtenborg classified more than 4,000 isolates of terverticillate penicillia into 38 taxa and chemotypes, where infrataxon strains exhibited chemoconsistency in terms of the production of mycotoxins [79]. They also reidentified a large number of misidentified *Penicillium* strains based on their profiles of secondary metabolites [[79], [80], [81]]. Frisvad and Filtenborg, together with Samson and Stolk, also pioneered the chemotaxonomy of *Talaromyces*. Again, their analysis demonstrated that the production of secondary metabolites by members of this genus was taxon-specific and they also recognised *T. macrosporus* and *T. luteus* as separate species from *T. flavus* and *T. udagawae*, respectively, because of their different metabolic profiles [42]. In fact, this chemotaxonomic work offered one of the very first indications of the connection between *Talaromyces* and *Penicillium* subgenus *Biverticillium* [42]. An overview of the extrolite profiles for various *Talaromyces* species was given in the latest monograph on the genus by Yilmaz et al. [63]. The same also applies to *Aspergillus* species [[82], [83], [84], [85], [86]]. Notably, different *Aspergillus* subgenera produce different unique extrolites, as summarised by Frisvad and Larsen [87]. Thus, the production of a certain secondary metabolite by an *Aspergillus* isolate would serve as a practical hint for identification at the sectional level, whereas the identification of several secondary metabolites of the organism would be an effective tool for species recognition [2]. Currently, (ultra) high-performance liquid chromatography (UHPLC/HPLC) coupled with diode array detection (DAD) and mass spectrometry (MS) is the method of choice for detailed chemotaxonomic characterisation of *Aspergillus*, *Penicillium* and *Talaromyces* [1,2,63,88]. With about 350 accepted species each in *Aspergillus* [2,60] and *Penicillium* [1] and more than 100 accepted species in *Talaromyces* [63,89], qualitative databases equipped with a large volume of verified data on the production of secondary metabolites by various *Aspergillus*, *Penicillium* and *Talaromyces* species is needed for accurate species identification [2]. In view of this, an *Aspergillus* Secondary Metabolites Database (A2MDB) was established last year [90]. Recently, metabolic fingerprinting has also been demonstrated as a potentially successful tool for differentiating closely related *Aspergillus* species, without the need of investigating the actual identities of the metabolites. For example, utilising this technique Tam et al. showed that *A. nomius* and *A. tamarii* could be distinguished from their morphologically similar sibling *A. flavus* [91]. In addition, hierarchical cluster analysis by Tsang et al. also showed that except for *A. austroafricanus*, the metabolic fingerprints of species in the same *Aspergillus* section clustered together and those of infraspecific strains also formed smaller subclades [92].

Fatty acid profiling is another increasingly used method in diagnosing filamentous fungal species. Although characterisation of fatty acid composition and relative concentration has long been utilised for bacterial and yeast chemotaxonomy [93,94] and there is even a commercial fatty acid methyl ester (FAME)-based bacterial/yeast identification system containing profiles from more than 1,500 different species developed [95], there are only a few studies making use of this technique to characterise the chemotaxonomy of filamentous fungi [96]. This is because filamentous fungi do not produce fatty acids in the quantity and diversity that bacteria do [97] and therefore, traditionally fatty acid profiling had been regarded to have little taxonomic value for filamentous fungi [98]. Blomquist et al. [99] first examined the utility of this technique on the identification of filamentous fungi. They characterised the fatty acid contents of conidia and found that fatty acid profiling, even though performed at different times, could potentially be used to identify *Aspergillus* and *Penicillium* species in a reproducible way [99]. In 1996, Stahl and Klug performed a large-scale study to characterise the composition and relative concentration of fatty acids in the mycelia of a number of filamentous fungi from across different phyla [98]. Seven species of *Penicillium* and one of *Aspergillus* were included in their study. It was revealed that four fatty acids, namely palmitic acid (C16:0), stearic acid (C18:0), oleic acid (C18:1Δ^9^[*cis*]) and linoleic acid (C18:2Δ^9,12^[*cis*]), represented more than 95% of the total cellular fatty acid content. These four fatty acids were also common to all the filamentous fungi characterised. In spite of this, discriminant analysis showed that the fatty acid profiles for these species are significantly different. Notably, all the seven *Penicillium* species characterised were found to possess unique fatty acid profiles [98]. Later in 1998, Da Silva et al. expanded the characterisation to 18 *Penicillium* species [100]; and they found that different *Penicillium* subgenera could be readily differentiated by fatty acid profiling. Moreover, in some cases, species of the same subgenus such as *Furcatum* could be separated based on their fatty acid profiles, which mainly differed in the relative concentration rather than the composition of fatty acids; although difficulties existed for the subgenus *Penicillium* [100]. The fact that the species differentiation power relied on the variation in fatty acid relative concentration was observed by Mahmoud et al. as well [101]. Fatty acid profiling has also been successfully used to differentiate *Aspergillus* species [102,103].

A recent chemotaxonomic approach for rapid identification of *Aspergillus*, *Penicillium* and *Talaromyces* is matrix-assisted laser desorption/ionisation–time-of-flight (MALDI–TOF) MS. The technology compares the cellular protein profiles of different organisms to achieve identification at the species level [104]. The advantage of this technique is that the methodology is simple, rapid and inexpensive, requiring a specialised bench-top MALDI–TOF mass spectrometer only. Also, since the majority of proteins analysed by MALDI–TOF MS are constitutively expressed ribosomal proteins, microorganisms can be successfully identified even though varying culture media and incubation conditions are used [104,105]. More importantly, databases consisting of protein mass spectra from over 2,400 microbial species are commercially available [106,107], making the identification of a wide range of microorganisms possible. Given its numerous advantages, MALDI–TOF MS has been gaining popularity for identification of pathogenic microorganisms, including bacteria [108,109], yeasts [[108], [109], [110], [111], [112], [113], [114], [115]] and even filamentous fungi [109,[116], [117], [118]], in clinical microbiology laboratories. The potential of this technology in diagnosing *Aspergillus*, *Penicillium* and *Talaromyces* species has also been evaluated by numerous studies. In general, MALDI–TOF MS is successful in identifying the more commonly found aspergilli/penicillia, such as *A. flavus*, *A. fumigatus*, *A. nidulans*, *A. niger*, *A. sydowii*, *A. unguis*, *P. chrysogenum*, *P. aurantiogriseum* and *P. purpurogenum*, with correct identification rates of ≥78% [[117], [118], [119], [120], [121]]. Yet, for other rare species misidentification is often encountered. Notably, these uncommon species could usually be identified to the sectional level. For example, *A. tritici* (section *Candidi*) was misidentified as *A. candidus*; *A. oryzae* (section *Flavi*) as *A. flavus*; *A. fischeri* (section *Fumigati*) as *A. fumigatus*; *A. tubingensis* and *A. welwitschiae* (section *Nigri*) as *A. niger*, *A. hortai* and *A. niveus* (section *Terrei*) as *A. terreus*; as well as *A. sydowii* (formerly section *Versicolores*) as *A. versicolor* [92,122]. A probable reason for this is that the mass spectra for many of these rare species are lacking in the commercial libraries. It should be noted that the Bruker MBT MSP 6903 Library, Bruker MBT Filamentous Fungi Library and Vitek MS V3.0 Knowledge Base only include reference mass spectra for 42, 127 and 82 filamentous fungal species, respectively [106,117,123]. Of these, only up to 22 *Aspergillus*, 21 *Penicillium* and 6 *Talaromyces*, which are still named with their previous *Penicillium* synonyms, species are included [107,123]. However, the numbers of accepted *Aspergillus*, *Penicillium* and *Talaromyces* species greatly outnumber those included in the MALDI–TOF MS databases, with both *Aspergillus* and *Penicillium* having approximately 350 species [1,2,60] and *Talaromyces* having more than 100 species [63,89]. Despite this, MALDI–TOF MS has still been demonstrated as a potential tool to differentiate members of the three genera by hierarchical cluster analysis of the mass spectra of various species [91,124,125]. As a result, theoretically if more reference mass spectra for different species, especially the rare ones, are generated for inclusion in the databases the species diagnosis power of MALDI–TOF MS would be greatly enhanced and it has already been exemplified by previous studies that the correct identification rates could be improved by the expansion of reference libraries using inhouse generated mass spectra [118,122,125]. To overcome the limitation of small reference data volume of the commercial databases, several organisations have self-established online supplementary databases. For example, the Spectra database (freely available at https://spectra.folkhalsomyndigheten.se/spectra/) by the Public Health Agency of Sweden (Folkhälsomyndigheten) is a platform for MALDI–TOF MS users to deposit and exchange user-generated mass spectra which are curated and continuously updated. Another such complementary database is the MSI Platforme which serves as a webtool for MALDI–TOF MS-based fungal identification. This platform contains more than 11,800 reference mass spectra of more than 900 fungal species, aiming at supplementing the insufficient spectral diversity of the commercial databases so as to improve species identification [126].

With the current adoption of consolidated species recognition where molecular characters play a predominant role, DNA sequencing and phylogenetic analysis have become the gold standard for accurate fungal identification. As in other fungi, early molecular work on *Aspergillus*, *Penicillium* and *Talaromyces* involved the comparison of large and small subunit ribosomal nucleic acid (mitochondrial and/or nuclear) as well as internal transcribed spacer (ITS) sequences [[43], [44], [45],^127^]. However, subsequent analysis showed that ribosomal genes are too conserved to separate these groups of fungi [128,129]. In addition, although ITS is now accepted as the official DNA barcode for fungi [130], it has also been recognised as an extremely conserved region for *Aspergillus*, *Penicillium* and *Talaromyces* [1,2,63]. Despite the fact that its sequence variability could be used to distinguish species belonging to different sections or series [128], very often it is not useful for the differentiation of species within the same section or series. In view of this and also to better reflect the genealogy of this group of organisms, sequencing of multiple genetic markers, in particular the β-tubulin (*benA*) and calmodulin (*cmdA* or *CaM*) genes, to define species boundaries has been advocated [131]. The exons of these genes are highly conserved and are therefore good locations for primer binding, whereas introns in between the exons act as the major source of sequence variation. As a result, sequences of these genes containing both exons and introns are able to provide variations at different levels for species delimitation [131]. With the majority of *Aspergillus*, *Penicillium* and *Talaromyces* species clearly defined nowadays, sequencing of *benA* and/or *cmdA* can be utilised to identify most of these species. In fact, *benA* and *cmdA* have been proposed as the secondary identification markers for *Penicillium* and *Aspergillus* species, respectively [1,2]. This is because there are universal primers available for these two genes and both of them are easy to amplify. In the case of *Aspergillus*, although *benA* could be easily amplified, the presence of paralogous genes (e.g. *tubC*) in some species which could also be amplified by the universal primers could be confusing and complicate species identification [132,133]. In contrast, although a similar problem has also been noted for *cmdA*, amplification of a pseudogene only occurred for one *Aspergillus* strain [134]. Moreover, *cmdA* is also easy to amplify and its sequence is available for nearly all accepted species. Therefore, *cmdA* was chosen over *benA* as secondary identification marker for *Aspergillus* [2]. On the other hand, as for *Penicillium*, amplification of *benA* paralogues has not been reported and since a complete *cmdA* sequence database is lacking, *benA* became the secondary identification marker of choice [1]. Although a third option, RNA polymerase II second-largest subunit gene (*rpb2*), also exists and its lack of introns allows robust and easy alignment for phylogenetic analysis, it was not selected over *benA* or *cmdA* because *rpb2* is sometimes difficult to amplify and a database with sufficient volume is lacking [1,2]. Nonetheless, when resources are available it is recommended to sequence all the four genetic markers (ITS, *benA*, *cmdA* and *rpb2*) to aid identification, especially when new species are diagnosed [1,2]. Although a recommendation of identification markers has not been put forward for *Talaromyces* species, they generally follow those for *Aspergillus* and *Penicillium* species [63]. In order to achieve accurate identification, sequences from reliable databases should be compared against. Despite the fact that the International Nucleotide Sequence Database Collaboration (INSDC) [135] contains a vast number of sequences, the reliability of the sequence annotation is questionable [136,137]. Notably, ≥10% of the fungal ITS sequences in these databases were found to be misannotated [136]. As such, the Fungal ITS RefSeq Targeted Loci Project has been initiated by the National Center for Biotechnology Information (NCBI) to improve the quality and accuracy of the sequences deposited to INSDC [138,139]. Similarly, the UNITE database was developed to include high-quality type or representative sequences for fungi or fungal species hypothesis with correct or up-to-date taxonomic annotations [140]. The International Society for Human and Animal Mycology (ISHAM) ITS database, specialised in the ITS-based identification of medical fungi, has also been recently established [141] and it contains quite a number of high-quality ITS sequences for *Aspergillus*, *Penicillium* and *Talaromyces* species, which are commonly encountered in the clinical settings. While curated databases for *benA*, *cmdA* and *rpb2* have not been created, reliable sequences for all the ex-type strains of *Aspergillus*, *Penicillium* and *Talaromyces* accepted species have been listed in the recent monographs on the three genera [1,2,63] or online at http://www.aspergilluspenicillium.org/. In addition to nuclear genes, attempts have also been made to understand the evolution (and thus species recognition) of *Aspergillus*, *Penicillium* and *Talaromyces* by sequencing of mitogenomes [[142], [143], [144], [145]]. Yet, only a handful of mitogenomes are available for these groups of fungi currently and the utility of mitogenomes for species diagnosis awaits further examination.

## 4 Clinical perspectives

A stable taxonomy is important to the study of *Aspergillus*, *Penicillium* and *Talaromyces* in every aspect including medical mycology. First of all, the nomenclature of pathogenic fungi should be steady over time, without frequent vigorous name changes. The recently implemented 1F1N scheme, where one fungus shall only possess one name, drastically simplified fungal nomenclature. The accepted use of *Aspergillus* and *Penicillium* names over their respective ‘sexual names’ is particularly important to the medical community. This is because most clinical fungi are isolated in the asexual forms and these fungi are traditionally named with their asexual names. Use of the ‘sexual names’ would confuse clinicians since they would not be aware of what *Eupenicillium*, *Neosartorya* and *Emericella* are, thus hindering treatment and patient care. This could be exemplified by the recent transfer of *P. marneffei* to *T. marneffei*, where the well-known disease name ‘penicilliosis’ also has to be changed to the unfamiliar ‘talaromycosis’. A stable taxonomy also clearly defines species and their identification methods. Therefore, the clinical spectrum of pathogenic species could also be better studied. In particular, rare and new aetiological agents could be revealed (Table 2) [92,[146], [147], [148], [149], [150], [151]]. Accurate identification of the causative pathogen is crucial to epidemiological studies. Correct species diagnosis could also help predict antifungal susceptibility, which varies across different species and this could significantly affect patient treatment, disease management and prognosis. For example, it has been shown that *A. tubingensis* and *A. unguis* possessed elevated minimum inhibitory concentrations (MICs) to itraconazole [92]. The fact that triazole agents exhibit various activities against different *Aspergillus* species has also been demonstrated by other studies [148,149,151]. Also, although triazoles showed moderate activities against *Penicillium* species, their effectiveness against some *Talaromyces* species are poor [147].

**Table 2:** Novel *Aspergillus*, *Penicillium* and *Talaromyces* species/taxonomic entities described during January, 2013 to December, 2017 sampled from human or non-human vertebrate specimens

Species	Synonym(s)	Associated human infections or clinical specimens^a^	Associated non-human vertebrates	Molecular markers^b^	Year of valid publication	Reference(s)
*Aspergillus*						
*A. aurantiopurpureus*	Novel species		Kangaroo rat cheek pouch	ITS, *benA, cmdA* and *rpb2*	2016	[86]
*A. caninus*	≡ *Phialosimplex caninus,*		Bone marrow aspirate of a dog	*rpb2*	2014	[2,176,177]
*A. capsici*	≡ *Scopulariopsis capsica*		Fur and skin of hibernating bat		2014	[2,178]
	= *Leuconeurospora capsica*					
*A. chlamydosporus*	≡ *Sagenomella chlamydospore*		Disseminated infection in a dog	*rpb2*	2014	[2,176,179,180]
	= *Phialosimplex chlamydosporus*					
*A. citrinoterreus*	Novel species	Nails, various respiratory specimen, wound and biopsy		*benA* and *cmdA*	2015	[181]
*A. contaminans*	Novel species	Fingernail (probably as a contaminant)		ITS, *benA*, *cmdA* and *rpb2*	2017	[182]
*A. europaeus*	Novel species	Toenail		ITS, *benA* and *cmdA*	2016	[183]
*A. felis*	Novel species	Chronic invasive pulmonary aspergillosis and onychomycosis; BAL, oropharyngeal exudate and sputum	Invasive fungal rhinosinusitis in domestic cats and disseminated invasive aspergillosis in a dog	ITS, *benA* and *cmdA*	2013	[[184], [185], [186], [187]]
*A. hongkongensis*	Novel species	Onychomycosis		ITS, *benA*, *cmdA*, *rpb2*, *mcm7* and *tsr1*	2016	[92]
*A. insolitus*	≡ *Polypaecilum insolitum*	Onychomycosis; ear		*cct8*, *rpb2* and *tsr1*	2014	[2,188]
*A. keratitidis*	≡ *Sagenomella keratitidis*	Keratitis		ITS and 28S nrDNA	2017	[189,190]
*A. latilabiatus*	Novel species		Sheep dung	ITS, *benA*, *cmdA* and *rpb2*	2016	[86]
*A. latus*	≡ *Aspergillus nidulans* var. *latus*	Invasive pulmonary aspergillosis		ITS, *benA*, *cmdA* and *rpb2*	2016	[53,86,[191], [192], [193], [194], [195]]
	= *Aspergillus montenegroi*					
	= *Aspergillus sublatus*					
	= *Emericella montenegroi*					
	= *Emericella nidulans* var. *lata*					
	= *Emericella sublata*					
*A. magnivesiculatus*	Novel species	Child carriers		ITS, *benA*, *cmdA* and *rpb2*	2017	[171]
*A. mallochii*	Novel species		Pack rat dung	*benA*, *cmdA* and *rpb2*	2017	[196]
*A. microperforatus*	Novel species	Lymph node and toenail		ITS, *benA*, *cmdA* and *rpb2*	2017	[151]
*A. pallidofulvus*	≡ *Aspergillus sulphureus* var. *minimus*	Invasive pulmonary aspergillosis and disseminated aspergillosis		ITS, *benA*, *cmdA*	2014	[122,197]
*A. parafelis*	Novel species	Invasive aspergillosis; oropharyngeal exudate and sputum	Cats	*benA*, *cmdA, rpb2*, *mcm7* and *tsr1*	2014	[198,199]
*A. pragensis*	*Aspergillus* section *Candidi*	Onychomycosis		ITS, *benA* and *cmdA*	2014	[200]
*A. pseudofelis*	Novel species	Invasive aspergillosis; sputum and nail		*benA*, *cmdA, rpb2*, *mcm7* and *tsr1*	2014	[198]
*A. pseudogracilis*	Novel species	Child carrier		ITS, *benA*, *cmdA* and *rpb2*	2017	[171]
*A. pseudosclerotiorum*	Novel species	BAL, lung and sputum		ITS, *benA*, *cmdA* and *rpb2*	2017	[149]
*A. pseudoviridinutans*	Novel species	Invasive aspergillosis; mediastinal lymph node		*benA*, *cmdA*, *rpb2*, *mcm7* and *tsr1*	2014	[198]
*A. reticulatus*	Novel species	Lung biopsy, child carrier		ITS, *benA*, *cmdA* and *rpb2*	2017	[171]
*A. sclerotialis*	≡ *Sagenomella sclerotialis*		Dog	*rpb2*	2014	[2,176,201]
	= *Phialosimplex sclerotialis*					
*A. spinulosporus*	≡ *Aspergillus nidulans* var. *echinulatus*	Recurrent prosthetic valve endocarditis and invasive pulmonary aspergillosis		ITS, *benA, cmdA* and *rpb2*	2016	[2,[202], [203], [204], [205], [206], [207], [208]]
	= *Aspergillus delacroixii* (Samson, Visagie & Houbraken)					
	= *Emericella echinulate*					
	= *Emericella nidulans* var. *echinulata*					
*A. stercorarius*	Novel species		Lizard (*Uromastix acanthinurus*) dung	ITS, *benA*, *cmdA* and *rpb2*	2016	[86]
*A. wyomingensis*	Novel species		Cat	*benA* and *cmdA*	2014	[134,209]

*Penicillium*						
*P. canis*	Novel species		Bone lesion of spayed Rhodesian ridgeback dog with osteomyelitis	ITS, *benA* and *cmdA*	2014	[210]
*P. fimorum*	Novel species		Mouse dung	ITS, *benA*, *cmdA* and *rpb2*	2016	[173]
*P. paradoxum*	*≡ Aspergillus paradoxus*		Dung of dog and opossum	ITS, *benA*, *cmdA* and *rpb2*	2014	[1,202,211,212]
	*= Aspergillus ingratus*					
	*= Hemicarpenteles paradoxus*					
*P. robsamsonii*	Novel species		Mouse dung	ITS, *benA*, *cmdA* and *rpb2*	2016	[173]

*Talaromyces*						
*T. alveolaris*	Novel species	BAL		ITS, *benA*, *cmdA* and *rpb2*	2017	[150]
*T. atroroseus*	Novel species		Mouse dung	ITS, *benA* and *rpb1*	2013	[213]
*T. columbinus*	Novel species	Fungaemia and pulmonary nodule and adjacent rib osteomyelitis		ITS, *benA*, *cmdA*, *rpb1*, *rpb2*, *mcm7* and *tsr1*	2013	[[214], [215], [216]]
*T. georgiensis*	Novel species		Animal joint fluid	ITS, *benA* and *rpb2*	2017	[150]
*T. kabodanensis*	Novel species	BAL		ITS, *benA*, *cmdA* and *rpb2*	2016	[150,217]
*T. minnesotensis*	Novel species	Ear		ITS, *benA*, *cmdA* and *rpb2*	2017	[150]
*T. rapidus*	Novel species	BAL		ITS, *benA*, *cmdA* and *rpb2*	2017	[150]
*T. siglerae*	Novel species	Tinea capitis		ITS, *benA*, *cmdA* and *rpb2*	2017	[218]

a BAL, bronchoalveolar lavage

b *benA*, β-tubulin gene; *cct8*, chaperonin-containing T-complex protein 1 subunit theta gene; *cmdA*, calmodulin gene; ITS, internal transcribed spacer; *mcm7*, mini-chromosome maintenance complex component 7 gene; nrDNA, nuclear ribosomal rRNA gene; *rpb1*, RNA polymerase II largest subunit gene; *rpb2*, RNA polymerase II second largest subunit gene; *tsr1*, ribosome maturation factor for 20S rRNA accumulation gene

## 5 Summary and outlook

With a consistent taxonomy, understanding on the epidemiology and clinical spectrum of diseases caused by *Aspergillus*, *Penicillium* and *Talaromyces* could be enhanced. This in turn facilitates laboratory diagnosis of these important mycotic pathogens and establishment of patient treatment strategies. The transition from morphological/phenotypic to chemotaxonomic, genetic/phylogenetic, or consolidated species recognition results in the reclassification of these groups of fungi and enables sexual-asexual connection. In the current omics era, advancement in different omics technologies makes characterisation of the complete set of a particular group of characters possible, allowing more thorough analyses and therefore, a more stable taxonomy. For example, comparison of mitogenomes supported the transfer of ‘*P. marneffei*’ to *Talaromyces* and demonstrated that *Aspergillus* and *Penicillium* are more closely related to each other than to *Talaromyces* [142,144,145]. The availability of contemporary advanced techniques, such as MALDI–TOF MS as well as UHPLC/HPLC–DAD–MS, significantly improves proteomic and metabolic fingerprinting of fungi, respectively, thus aiding chemotaxonomy. As the cost for second-generation sequencing is getting lower and the emerging third-generation sequencing is becoming more widely accessible, more and more complete/almost complete fungal genomes become available. These genome sequences could advance our knowledge on these fungi, such as *T. marneffei* [144,[152], [153], [154], [155], [156], [157], [158]], and taxonomy on them could thus be facilitated. With such additional novel data, further reclassification on *Aspergillus, Penicillium* and *Talaromyces* is expected. Application of all these state-of-the-art omics technologies is likely to provide comprehensive information on the evolution of the three related genera, and a more stable taxonomy for them will hopefully be achieved. Yet, it should be noted that even though these advanced methodologies are becoming more readily available for the identification and classification of fungi, it is equally important for mycologists to apply standard or best practices when studying fungal taxonomic relationships. In particular, fungal taxonomists should always keep themselves up-to-date with recent trends, tools, standards, recommendations and practices in the field, especially when describing new species [[159], [160], [161], [162], [163]]. When depositing DNA sequence data to public databases, the sequences should be well checked for authenticity as well as reliability [164], and they should be richly annotated as far as possible [165]. Also, multiple genetic markers and proper analytical tools should be used for the inference of phylogenetic relationships [166]. As nowadays taxonomy has entered a deep crisis where descriptive taxonomic studies are not encouraged, it is important for taxonomists to keep the pace for re-growth, to participate actively and to form a good ‘taxonomic culture’ so that the scientific community would value taxonomic work higher [167]. This could also help attract more research funding for more expensive technology or equipment for more detailed taxonomic characterisation. All these efforts could help speed up taxonomic and molecular ecology progress on *Aspergillus*, *Penicillium* and *Talaromyces* significantly.
